# Sonoanatomy of the Posterior Cutaneous Nerve of the Forearm: A Proposed Approach for Blockade in Lateral Epicondylitis (LEPI-Block)

**DOI:** 10.7759/cureus.117983

**Published:** 2026-10-15

**Authors:** Thomas Helfenstein, Thadeu R Da Costa, Roberto D Rached

**Affiliations:** 1 Legal Medicine, Bioethics, Occupational Medicine, and Physical Medicine and Rehabilitation, Instituto de Medicina Fisica e Reabilitacao, Hospital das Clínicas da Faculdade de Medicina da Universidade de São Paulo (HCFMUSP), São Paulo, BRA; 2 Physical Medicine and Rehabilitation, Universidade de São Paulo, Hospital das Clínicas, São Paulo, BRA

**Keywords:** lateral epicondylitis, local analgesia, neuromodulation therapies, pain, ultrasound anatomy

## Abstract

Lateral epicondylitis (LE), commonly known as tennis elbow, is a prevalent cause of chronic lateral elbow pain and functional limitation. Despite a range of conservative and interventional treatments, many patients experience persistent symptoms, and there is no universally accepted gold standard therapy. This descriptive anatomical study aimed to establish an ultrasound-guided technique to identify and target the posterior cutaneous nerve of the forearm (PCNF), a sensory branch of the radial nerve implicated in lateral epicondylar innervation. Twenty elbows from healthy volunteers were evaluated using high-resolution ultrasound to visualize the PCNF and determine its anatomical relationship to the lateral epicondyle. A structure consistent with the PCNF was identified in all 20 ultrasound examinations, with a mean PCNF depth of 6.03 ± 0.26 mm from the skin surface and a mean distance of 9.40 ± 0.95 mm from the lateral epicondyle. In one case, hydrodissection followed by low-voltage sensory stimulation enabled functional sensory mapping, providing preliminary functional evidence supporting the sensory distribution of the PCNF in the lateral epicondylar region. These findings support the feasibility of ultrasound-guided identification and targeting of the PCNF using the proposed LEPI-Block technique and establish a standardized sonoanatomical approach that may serve as a foundation for future diagnostic studies in patients with LE.

## Introduction

Lateral epicondylitis (LE), commonly known as "tennis elbow," is one of the most prevalent musculoskeletal disorders affecting the upper limb [1]. It has an annual incidence of about three to 11 per 1,000 individuals and a general population prevalence of 1% to 3%, rising to 23% among manual laborers [1]. Peak prevalence is observed in individuals aged 45 to 54 years, with no consistent sex predominance, although some studies report a slightly higher risk in women [1,2].

Clinically, LE is characterized by pain and tenderness over the lateral epicondyle of the humerus, where the common extensor tendon complex originates. This region serves as the common origin for the extensor muscles of the forearm, which are primarily responsible for wrist and finger extension and play a fundamental role in grip strength and upper-limb function. Repetitive or forceful movements, particularly those involving eccentric loading, are well-established mechanical contributors to the onset and perpetuation of symptoms [1,3].

Although diagnostic imaging is not routinely required, musculoskeletal ultrasound is increasingly used to evaluate tendon pathology, guide interventions, and monitor treatment response. While most cases resolve with conservative management, chronic or recurrent LE often poses a therapeutic challenge [3]. Persistent pain and functional impairment can significantly impact patients’ quality of life and occupational performance [3].

There is no accepted gold standard for the treatment of chronic LE. Various conservative and interventional therapies have been proposed, including corticosteroid injections, platelet-rich plasma (PRP), hyaluronic acid, prolotherapy, acupuncture, and physical therapy [4]. Among these, peripheral nerve blocks using local anesthetics offer an underutilized yet potentially valuable strategy for preventive analgesia and neuromodulation [5,6]. They can also serve as adjuncts to other procedures by reducing procedure-related discomfort and mitigating central sensitization [5].

Although several anatomical and cadaveric studies suggest that the lateral epicondyle region is innervated by the posterior cutaneous nerve of the forearm (PCNF), a sensory branch of the radial nerve that courses beneath the brachioradialis muscle, there remains a lack of standardized, objective, and clinically applicable ultrasound-based techniques to reliably identify this nerve in vivo [2,6]. This limitation contributes to inconsistent use of targeted nerve blocks in managing LE, despite their potential clinical utility.

Previous cadaveric investigations have demonstrated the sonoanatomy of the PCNF and validated its anatomical course using ultrasound-guided perineural injections followed by cadaveric dissection [7]. Furthermore, Finneran and Sandhu previously described an ultrasound-guided PCNF block in healthy volunteers [8]. However, a standardized ultrasound-guided approach focused on the lateral epicondyle, incorporating quantitative anatomical measurements and functional sensory mapping using low-voltage electrical stimulation, remains insufficiently explored in chronic LE. This study addresses this gap by proposing the LEPI-Block technique.

In this descriptive anatomical study, the primary objectives were to assess the feasibility of standardized sonographic identification of the PCNF in 20 healthy elbows, describe the proposed ultrasound-guided PCNF block technique (LEPI-Block), and map the sensory territory associated with the PCNF using low-voltage electrical stimulation. We hypothesized that evaluating 20 elbows would provide reproducible anatomical parameters to facilitate the proposed intervention. The proposed LEPI-Block may optimize pain control strategies in patients with chronic LE and redefine the approach to minimally invasive pain management.

## Technical report

This descriptive anatomical study was approved by the Research Ethics Committees System/National Research Ethics Commission (approval number: 82662024.4.0000.0068). All healthy volunteers provided written informed consent prior to enrollment, in accordance with the Declaration of Helsinki.

Twenty elbows from healthy adult volunteers were evaluated using high-resolution musculoskeletal ultrasound to investigate the optimal visualization technique for the PCNF, which innervates the lateral epicondyle region.

Eligible participants were adults aged 18 years or older with no history of elbow pain, musculoskeletal disorders, or upper limb surgery. Exclusion criteria included any current or past history of elbow pain (lateral or medial), history of trauma or surgery to the upper limbs, or congenital absence of one or both elbows. All procedures were performed in a controlled outpatient environment under strict aseptic conditions. The skin of the examined limb was cleaned with 2% chlorhexidine solution, then sterile ultrasound gel was applied. The ultrasound examination was conducted using a Mindray DC8 ultrasound system (Shenzhen Mindray Bio-Medical Electronics Co., Ltd., Shenzhen, China) equipped with a 3-12 MHz linear transducer.

All ultrasound examinations and interventional procedures were performed by a board-certified physiatrist with more than 10 years of experience in musculoskeletal ultrasound and ultrasound-guided interventions who had previously performed more than 500 ultrasound-guided procedures involving the lateral elbow region.

Healthy volunteers were positioned in a standardized manner, either seated or supine, with the shoulder in neutral rotation, the elbow flexed at 90°, and the forearm fully supinated, with the palm resting on the examination table. This positioning facilitated optimal sonographic access to the lateral aspect of the elbow. The examination targeted key anatomical landmarks, including the brachialis, biceps brachii, brachioradialis muscles, lateral epicondyle, and the PCNF.

In all healthy volunteers, continuous proximal nerve tracking was performed from the ultrasound-identified structure to its origin at the radial nerve, together with dynamic assessment of the surrounding muscular, fascial, and soft tissue anatomy. For clarity and reproducibility, this manuscript presents only the final ultrasound window corresponding to the proposed procedural target.

Sonographic images were captured in transverse and longitudinal planes, focusing on the course of the PCNF as it passes beneath the brachioradialis muscle toward the lateral epicondyle. All images were stored securely without any patient-identifying information.

Twenty healthy volunteers were prospectively enrolled in this descriptive sonoanatomical study, and only the right elbow of each volunteer was examined, resulting in 20 independent ultrasound evaluations. The study population included 12 females and 8 males, with a mean age of 27.8 ± 2.6 years (range, 24-35 years) and a mean body mass index of 23.8 ± 2.6 kg/m² (range, 19.2-28.6 kg/m²), as shown in Table 1. The dominant upper limb was not recorded because all anatomical measurements were referenced to fixed osseous landmarks, specifically the lateral epicondyle, rather than functional characteristics of the upper limb.

**Table 1:** Demographic characteristics of the healthy volunteers Demographic characteristics of the healthy volunteers included in the study (n = 20). Continuous variables are presented as mean ± SD, with the corresponding range in parentheses. Categorical variables are presented as numbers (percentages). SD: standard deviation

Variable	Unit	Value
Healthy volunteers	n	20
Examined elbows	n	20
Examined side	-	Right (100%)
Female	n (%)	12 (60%)
Male	n (%)	8 (40%)
Age	years	27.8 ± 2.6 (24-35)
Body mass index	kg/m²	23.8 ± 2.6 (19.2-28.6)

In one volunteer, ultrasound-guided hydrodissection of the PCNF was performed using a total volume of 3 mL of 5% dextrose solution, injected into the perineural tissue plane to separate the nerve from adjacent connective tissue structures and improve sonographic delineation. The injectate spread around the nerve, producing visible separation from the surrounding tissues under ultrasound guidance. Immediately following hydrodissection, without a waiting interval, functional sensory mapping was performed using a 22-gauge, 50-mm straight radiofrequency needle with a 4-mm active tip (Target Healthcare Dispositivos, Brazil) connected to a Zeek Medical Device radiofrequency generator. Under continuous ultrasound guidance, the needle tip was positioned immediately adjacent to the PCNF. The dispersive grounding electrode was placed over the ipsilateral posterior arm. No local anesthetic was administered for skin infiltration or elsewhere during the procedure to avoid interference with the sensory responses elicited by electrical stimulation.

Motor stimulation was performed before sensory stimulation at 2 Hz with a pulse width of 1 ms. The voltage was gradually increased from 0.1 to 0.8 V without eliciting any visible muscle contraction. Subsequently, sensory stimulation was performed with gradual voltage adjustment, and 0.4 V was identified as the lowest voltage eliciting a sensory response. The radiofrequency generator was used exclusively for diagnostic electrical stimulation and functional sensory mapping, without delivery of continuous or pulsed therapeutic radiofrequency energy.

Sensory stimulation was then applied at 50 Hz, a pulse width of 1.0 ms, and a maximum output of 0.4 V, sufficient to elicit the expected sensory perception without requiring voltage escalation. The volunteer continuously reported the location of the perceived sensation, and the corresponding cutaneous territory was immediately outlined on the skin with a dermatographic marker, allowing photographic documentation of the mapped sensory area.

This optional invasive component was performed in a single healthy volunteer who voluntarily agreed to undergo the procedure after receiving detailed information regarding its objectives, potential risks, and expected outcomes, and after providing additional written informed consent. Immediately after the procedure, the principal investigator performed a focused neurological examination, including assessment of superficial sensation within the mapped cutaneous territory, upper-limb muscle strength, and the presence of pain or other neurological abnormalities. This volunteer was subsequently followed prospectively with scheduled clinical evaluations every three months for two years. At each follow-up visit, the same neurological examination was repeated, and predefined adverse events, including pain, sensory disturbances (hypoesthesia, dysesthesia, or paresthesia), motor weakness, hematoma, infection, and any other procedure-related neurological or local complications, were systematically assessed and recorded.

All data were anonymized for analysis. The anatomical and procedural findings were compiled to develop a reproducible, step-by-step ultrasound-guided nerve block protocol, herein referred to as the proposed LEPI-Block.

Results

Ultrasound examinations were performed with the healthy volunteers positioned with the elbow flexed at 90°, the forearm fully supinated, and the palm resting comfortably on the examination table, as illustrated in Figure 1. The examiner faced the volunteer directly, and the ultrasound transducer was placed with its lateral edge over the lateral epicondyle. In some examinations, minor craniocaudal and mediolateral transducer adjustments, as illustrated in Figure 1, were required to obtain the standardized sonographic view shown in Figure 2.

**Figure 1:**
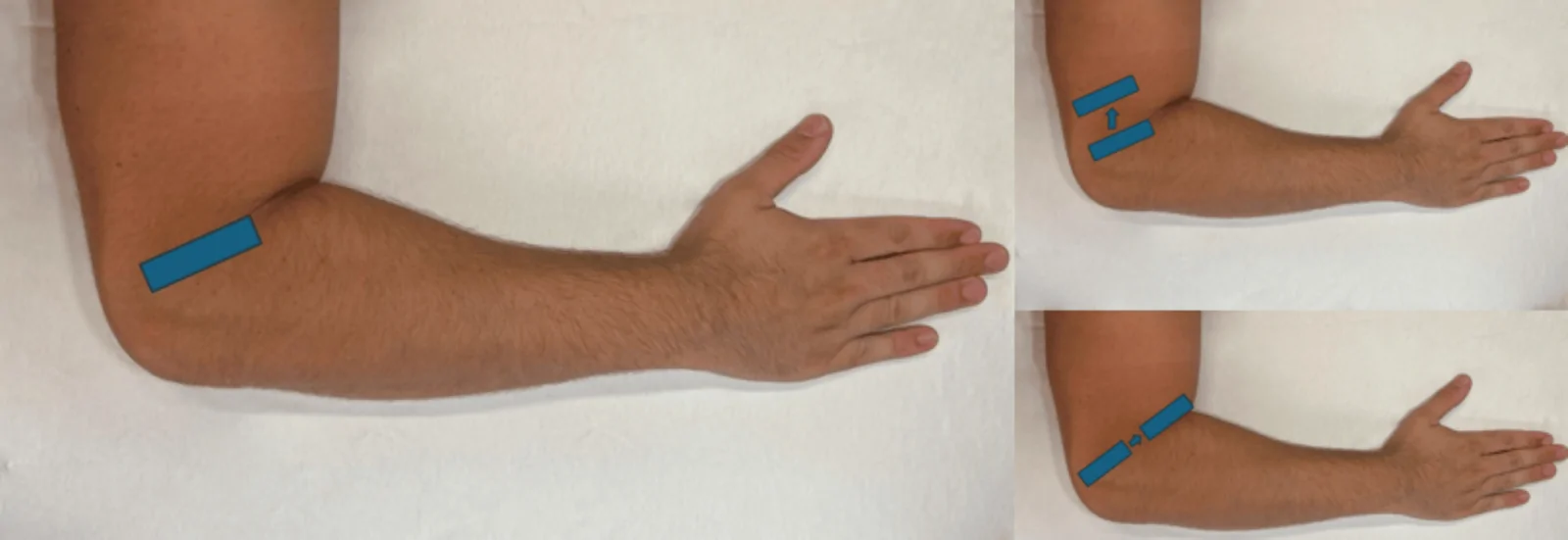
Ultrasound transducer positioning and adjustment The blue rectangle indicates the position of the ultrasound transducer; the blue arrow indicates its direction of movement.

**Figure 2:**
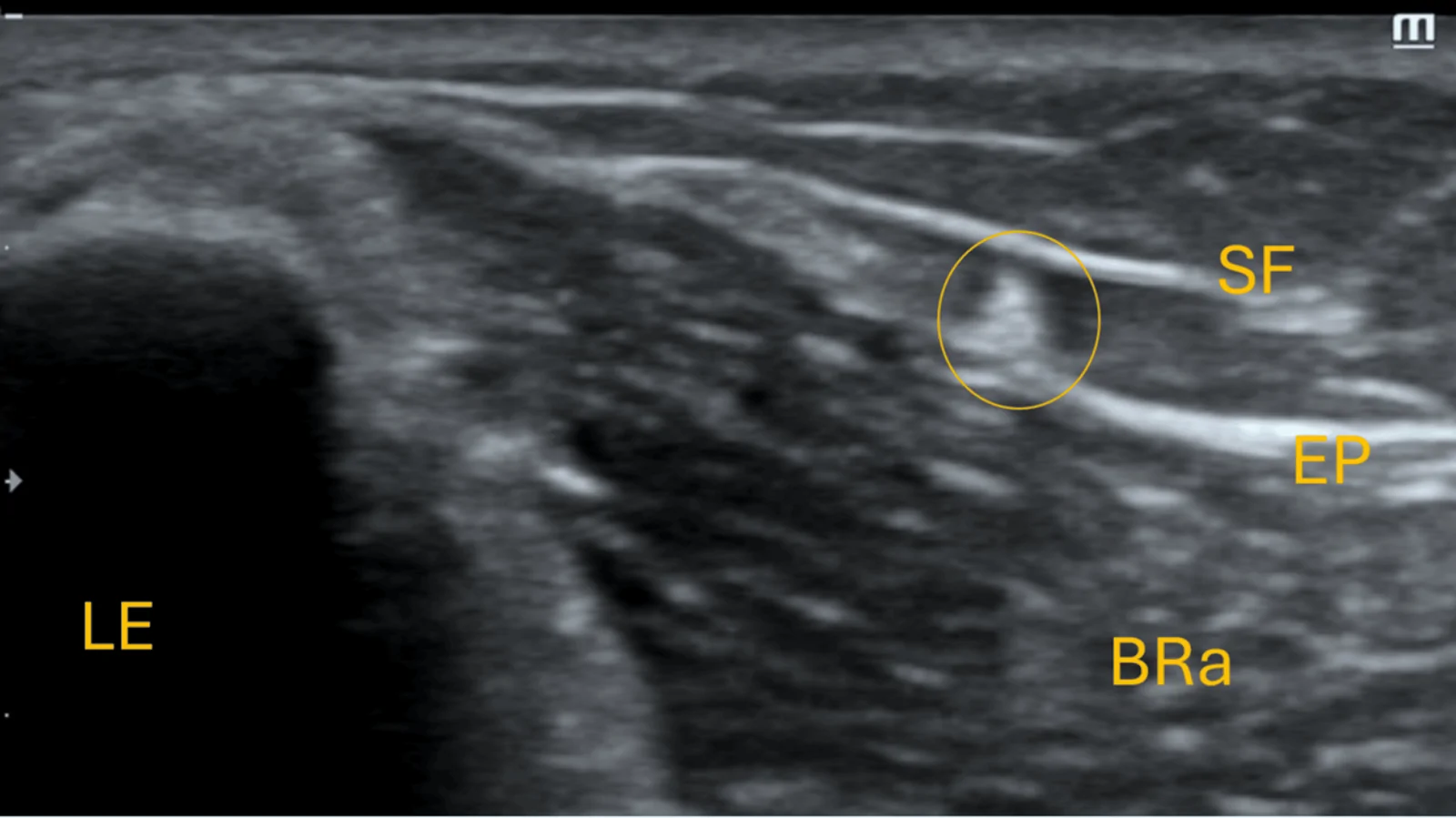
Ultrasound identification of the PCNF The yellow circle indicates the PCNF. LE: lateral epicondyle, BRa: brachioradialis muscle, SF: superficial fascia, EP: brachioradialis epimysium, PCNF: posterior cutaneous nerve of the forearm

In the reproducible sonographic plane (Figure 2), the skin and subcutaneous fat are visualized superficially, while the lateral epicondyle and its acoustic shadow serve as the lateral anatomical landmark. The brachioradialis muscle is identified in the deeper and medial portion of the image. In this plane, the PCNF, highlighted by the yellow circle, is visualized crossing the superior portion of the brachioradialis muscle.

In all 20 healthy volunteers, continuous proximal-to-distal ultrasound tracking showed that the PCNF branched from the radial nerve in the distal lateral arm and followed a distal course toward the lateral epicondylar region. At the proposed LEPI-Block level, the PCNF was consistently identified superficial to the brachioradialis muscle, within the fascial plane between the superficial fascia and the brachioradialis epimysium, without an intramuscular course. This anatomical relationship was observed in every examination.

Color Doppler imaging was performed during all ultrasound examinations to assess vascular structures along the proposed needle trajectory. No blood vessels were identified along this trajectory in any of the healthy volunteers.

Based on the measurements obtained from this standardized transverse sonographic plane, with the lateral epicondyle and PCNF simultaneously visualized, each anatomical parameter was measured once for each elbow, resulting in 20 individual measurements. The depth of the PCNF was measured linearly from the skin surface to the nerve, while the nerve-to-lateral-epicondyle distance was determined by a straight-line measurement using the adjacent cortical surface of the lateral epicondyle as the anatomical reference. The mean PCNF depth from the skin surface was 6.03 ± 0.26 mm, and the mean nerve-to-lateral-epicondyle distance was 9.40 ± 0.95 mm, both expressed as mean ± standard deviation. During image acquisition, minimal transducer pressure was applied to avoid compression of the superficial soft tissues and preserve the native anatomical relationship of the nerve. All measurements were subsequently performed offline on stored ultrasound images using the ultrasound system's electronic calipers.

In one healthy volunteer, using an in-plane, real-time ultrasound-guided approach, the needle was advanced from medial to lateral toward the ultrasound-identified PCNF (Figures 3-4). In the present study, hydrodissection was performed using 5% dextrose solution to temporarily separate the nerve from the surrounding connective tissues, thereby improving sonographic visualization and allowing subsequent functional sensory mapping with low-voltage electrical stimulation. Sensory stimulation was performed at 50 Hz, with a pulse duration of 1.0 ms and an intensity of 0.4 V. The measured impedance was 489 Ω, and the displayed temperature was 25°C.

**Figure 3:**
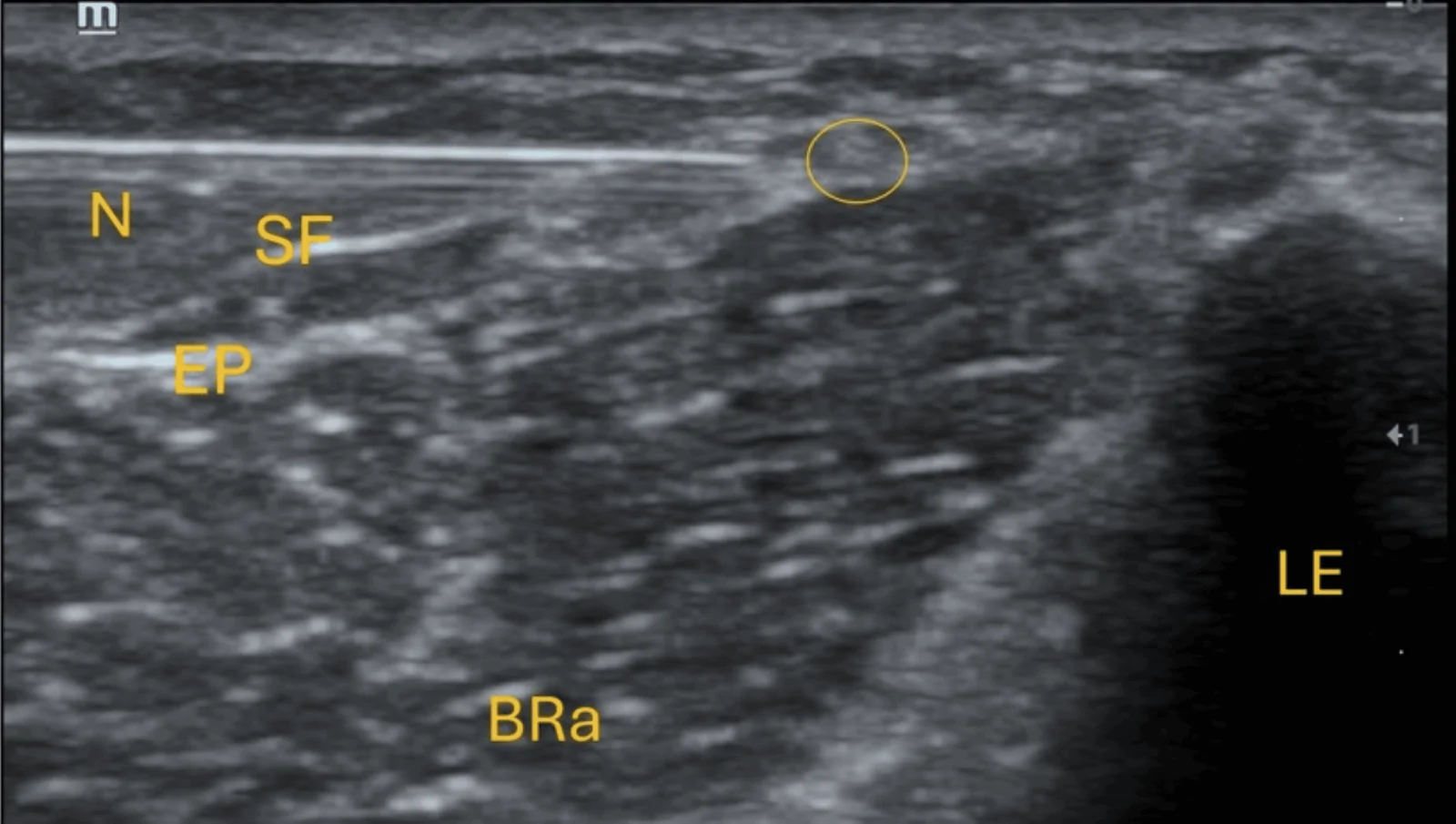
Ultrasound-guided needle approach to the PCNF The yellow circle indicates the PCNF. LE: lateral epicondyle, BRa: brachioradialis muscle, N: needle, SF: superficial fascia, EP: brachioradialis epimysium, PCNF: posterior cutaneous nerve of the forearm

**Figure 4:**
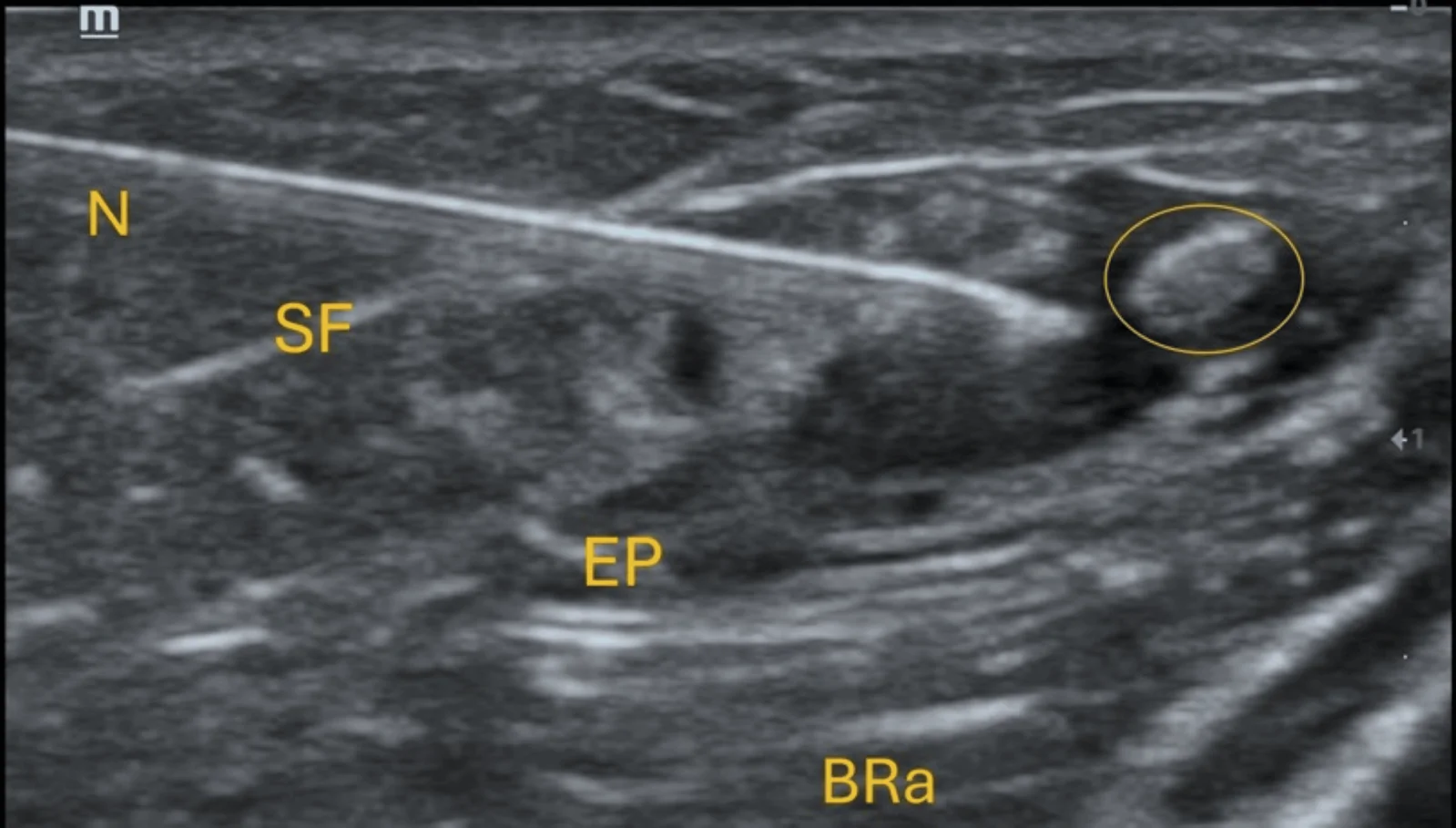
Ultrasound-guided hydrodissection of the PCNF The yellow circle indicates the PCNF. Hydrodissection of the PCNF showing hypoechoic fluid surrounding the nerve. LE: lateral epicondyle, BRa: brachioradialis muscle, N: needle, SF: superficial fascia, EP: brachioradialis epimysium, PCNF: posterior cutaneous nerve of the forearm

As shown in Figure 5, sensory mapping was performed by progressively stimulating the PCNF and asking the healthy volunteer to report the precise location of perceived sensation. Based on this feedback, the examiner outlined the sensory territory directly on the skin, corresponding to the areas identified by the volunteer as most sensitive.

**Figure 5:**
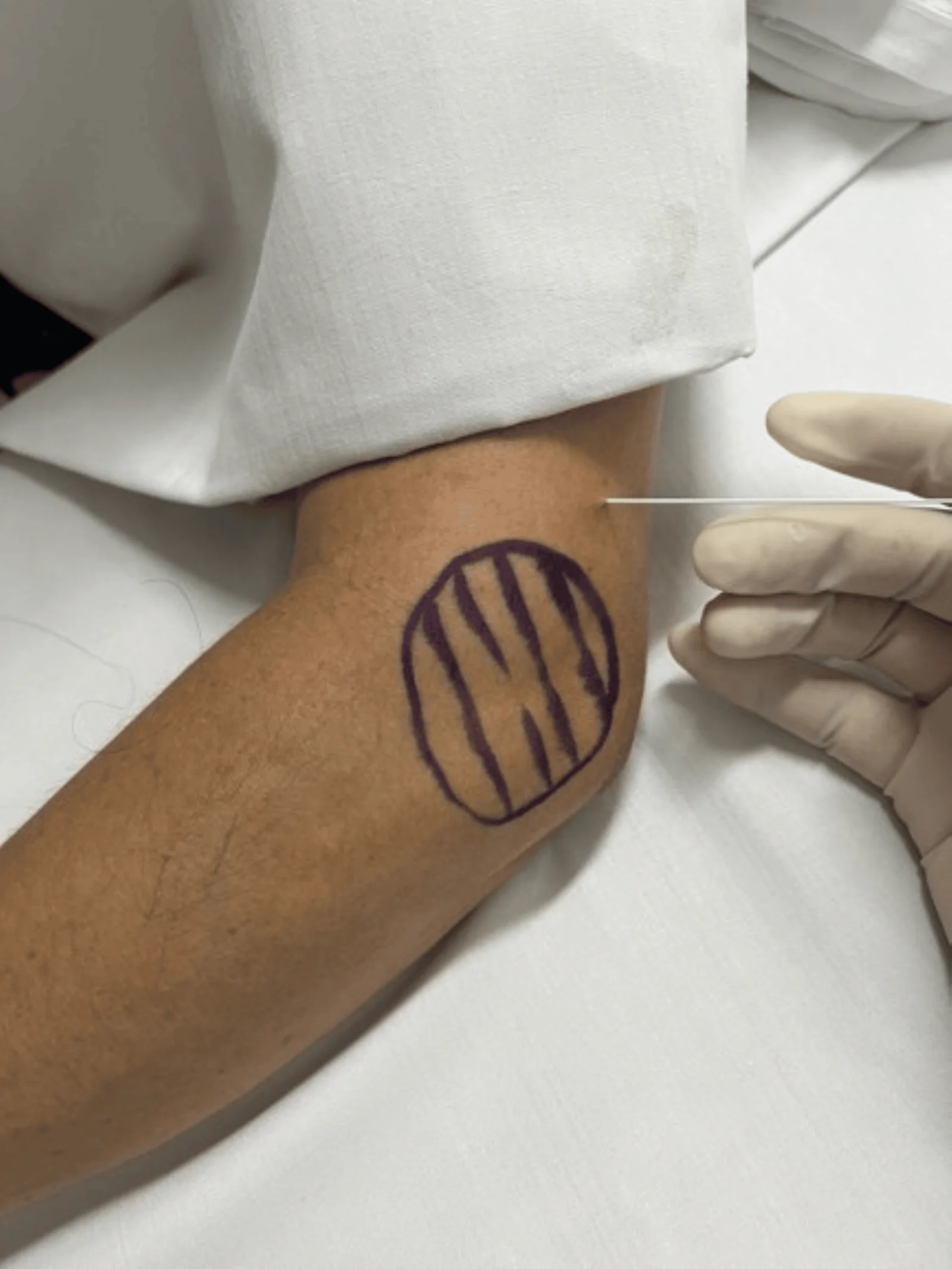
Sensory territory mapping following electrical stimulation of the PCNF Delineation of the sensitive area reported by the healthy volunteer following sensory stimulation. PCNF: posterior cutaneous nerve of the forearm

No adverse events were observed during or after the 20 ultrasound examinations. The study was conducted in accordance with all safety and ethical standards, under aseptic conditions and with full adherence to the approved research protocol.

The volunteer who underwent hydrodissection and sensory stimulation demonstrated no immediate neurological abnormalities on post-procedural examination. Throughout the scheduled two-year follow-up, no sensory deficits, motor weakness, pain, infection, hematoma, or any other procedure-related complications were observed.

## Discussion

LE is commonly managed initially through conservative measures aimed at reducing mechanical overload and inflammation [9]. These include modifying repetitive activities, temporarily suspending aggravating movements, using oral analgesics and nonsteroidal anti-inflammatory drugs when not contraindicated, applying ice after exertion, and physical therapy focused on wrist mobility and eccentric strengthening exercises [2,9]. Despite these strategies, pain may become chronic in a substantial number of cases, requiring further diagnostic and therapeutic interventions [9,10].

In persistent or diagnostically uncertain cases, imaging may be useful to confirm the diagnosis and rule out other causes of lateral elbow pain. Once LE is confirmed, several treatment options are available, although their levels of evidence and long-term efficacy vary [10]. Glucocorticoid injections have been widely used to relieve acute symptoms, but systematic reviews suggest they may worsen long-term outcomes by increasing recurrence rates [10]. PRP therapy, which aims to promote tissue repair via growth factors, has not consistently shown benefits in randomized trials for chronic LE [4]. Other minimally invasive procedures, such as acupuncture, percutaneous needle tenotomy, and extracorporeal shockwave therapy, have been used but lack sufficient high-quality evidence to establish strong recommendations [10].

Chronic lateral elbow pain is a heterogeneous clinical condition that may involve common extensor tendinopathy, lateral epicondylar enthesopathy, fascial or epimysial abnormalities, radiocapitellar joint pathology, synovial plica, lateral collateral ligament dysfunction, radial tunnel syndrome, posterior interosseous nerve involvement, cervical referred pain, and peripheral or central sensitization. Therefore, the proposed ultrasound-guided targeting of the PCNF should not be interpreted as a universal treatment for LE. Rather, it represents a potential regional sensory target that may warrant investigation in carefully selected patients after appropriate evaluation of other potential pain generators. This rationale is consistent with the well-established use of peripheral sensory nerves as therapeutic targets in several musculoskeletal pain conditions.

Future studies should determine whether the potential clinical effects of the proposed LEPI-Block arise predominantly from selective neural modulation, mechanical hydrodissection, fascial modulation, injectate spread toward the common extensor origin and lateral epicondylar enthesis, or a combination of these mechanisms. Although the PCNF was successfully identified in all 20 noninvasive ultrasound examinations, hydrodissection and electrical sensory stimulation were performed in only one healthy volunteer, without observed adverse events. The standardized sonoanatomical approach may provide a basis for future investigations of potential interventions, including anesthetic nerve blockade, neuromodulation, and denervation. The same ultrasound-guided needle trajectory and anatomical target may also be used for future perineural injection of a local anesthetic rather than 5% dextrose solution when performing the proposed LEPI-Block.

In this context, selective ultrasound-guided targeting of peripheral sensory nerves has emerged as a promising adjunctive approach, particularly in chronic musculoskeletal pain conditions. Whether this concept can be successfully applied to the PCNF in patients with LE remains to be established in future clinical studies. Local anesthetic blocks may provide preventive analgesia by modulating peripheral nociceptive input and potentially reducing sensitization, particularly when used before or alongside other potentially painful procedures. Infiltrative techniques involving corticosteroids, PRP, hyaluronic acid, prolotherapy, or dry needling often provoke nociceptive stimuli during or after administration [11]. When not accompanied by adequate anesthesia, these interventions may cause discomfort and, in some cases, worsen the pain experience. Therefore, the proposed LEPI-Block may represent a standardized ultrasound-guided approach that could facilitate future diagnostic and therapeutic interventions targeting the PCNF. However, its clinical value remains to be established in well-designed clinical studies.

Persistent nociceptive input from injured or inflamed deep somatic structures contributes to neurochemical alterations, cortical reorganization, and the perpetuation of central sensitization phenomena [12]. In this respect, approaches that aim to interrupt nociceptive transmission, such as targeted peripheral nerve blocks, may be more effective in addressing chronic pain than strategies that solely modulate inflammatory pathways [11,12].

The present study should be interpreted as complementary to previous cadaveric investigations rather than as a replacement for anatomical validation. Previous studies demonstrated the sonographic appearance of the PCNF, and ultrasound-guided perineural injections followed by cadaveric dissection confirmed its anatomical course [7]. Building on these findings, our study focused on a different objective: developing a simplified, standardized ultrasound-guided protocol that facilitates nerve identification using reproducible anatomical landmarks and quantitative measurements.

Additionally, unlike cadaveric investigations, we incorporated exploratory functional sensory mapping of the main PCNF in a single healthy volunteer. The observed cutaneous sensory responses provided preliminary support for a sensory territory associated with the ultrasound-identified nerve, without establishing exclusive PCNF stimulation or confirming its contribution to nociception from deeper structures, such as the common extensor origin or lateral epicondylar enthesis.

This study integrates previously described anatomical knowledge into a standardized ultrasound-guided protocol in healthy volunteers, combining reproducible anatomical landmarks, quantitative measurements, and functional sensory mapping to support future diagnostic and interventional applications involving the PCNF.

The ultrasound images presented in this manuscript were intentionally simplified to emphasize the procedural target and facilitate reproduction of the proposed technique by clinicians with varying levels of musculoskeletal ultrasound experience. Nevertheless, complete proximal nerve tracking of the radial nerve remains essential before performing any ultrasound-guided intervention involving the PCNF.

A recent cadaveric study described a humerus-parallel ultrasound-guided injection directed to the posterior antebrachial cutaneous nerve (PABCN)-related fascial plane, demonstrating injectate distribution along the superficial fascial plane while simultaneously reaching the common extensor origin and the lateral epicondylar enthesis through anatomical dissection [13]. Although that study used the term "PABCN," it refers to the same anatomical nerve designated in the present manuscript as the PCNF, following the current Federative International Program for Anatomical Terminology nomenclature.

Unlike that approach, which intentionally targets a nerve-related fascial plane and multiple potential pain generators, the LEPI-Block is a more selective, ultrasound-guided approach directed specifically at the PCNF. Hydrodissection was used to temporarily separate the nerve from the surrounding connective tissues before low-voltage sensory stimulation, thereby improving the selectivity of functional mapping. Consequently, both techniques should be regarded as complementary rather than competing approaches: the cadaveric study provides anatomical validation of injectate distribution within the fascial plane, whereas the present study contributes a standardized, reproducible ultrasound-guided identification protocol together with preliminary functional evidence supporting the corresponding sensory territory in a healthy volunteer.

As with several contemporary ultrasound-guided regional anesthesia techniques, the term "LEPI-Block" is proposed to establish standardized nomenclature for the ultrasound-guided approach, not to imply established clinical efficacy. Standardized terminology facilitates scientific communication, improves reproducibility, and provides a consistent framework for future investigations evaluating the safety, injectate distribution, block selectivity, diagnostic accuracy, and clinical effectiveness of the proposed LEPI-Block in patients with LE.

## Conclusions

The proposed LEPI-Block provides a standardized, ultrasound-guided approach to target the PCNF based on prespecified sonoanatomical landmarks. Functional sensory mapping using low-voltage electrical stimulation provided preliminary evidence supporting the sensory territory of the ultrasound-identified PCNF, complementing the anatomical findings and supporting further clinical investigation of this technique in chronic LE.

As this study was conducted in healthy volunteers and functional sensory mapping was limited to a single participant, further studies in symptomatic patients are required. Clinical trials should evaluate the safety and effectiveness of the LEPI-Block in reducing pain, improving function, and determining its potential role in the management of chronic LE.
